# Effectiveness of scenario-based learning and augmented reality for nursing students’ attitudes and awareness toward climate change and sustainability

**DOI:** 10.1186/s12912-022-01023-9

**Published:** 2022-09-03

**Authors:** Carmen Álvarez-Nieto, Cristina Álvarez-García, Laura Parra-Anguita, Sebastián Sanz-Martos, Isabel M. López-Medina

**Affiliations:** Department of Nursing, Faculty of Health Sciences, University of Jaen, Jaen, Spain

**Keywords:** Attitudes, Awareness, Climate change, Nursing students, Scenario-based learning, Sustainability

## Abstract

**Background:**

Mainstreaming sustainable healthcare into the curricula of health professions is a key action to raise awareness and change attitudes. Therefore, the present study aimed to assess the contribution of scenario-based learning and augmented reality to the environmental awareness and attitudes toward climate change and sustainability among undergraduate nursing students.

**Methods:**

This study was designed as a time-series analysis. Undergraduate nursing students in their 3 years were introduced to sustainability and climate change in the context of healthcare through scenario-based learning sessions. Questionnaires were used to collect data on participants’ attitudes towards sustainability and climate change, the usefulness of the educational sessions and the extent to which students changed their clinical practice. A data summary, related sample Friedman and Wilcoxon signed-rank tests were used to test for differences in survey scores.

**Results:**

Attitudes and environmental awareness toward climate change and sustainability increased significantly as students received the learning sessions over the 3 years. After their first clinical training period, students already showed a high awareness of unsustainable practices in their working environment; however, they still struggled to apply sustainability and address unsustainable practices in healthcare settings. Most students felt that the scenarios helped them to make links between climate change, resources, and health.

**Conclusions:**

The scenario-based learning and augmented reality increase environmental awareness and attitudes toward climate change and sustainability among nursing students. Students are very aware of unsustainable practices in their work environment, but more work needs to be done on the application of sustainability principles to nursing practice.

**Supplementary Information:**

The online version contains supplementary material available at 10.1186/s12912-022-01023-9.

## Background

The current pandemic provides further validation that creating a healthier, fairer, and greener world will be needed to resuscitate economies hit by the effects of COVID-19 [1, 2]. The effects of global warming and climate change have the potential to affect public health adversely, and are emerging as important and urgent public health problems [3]. The reality of climate change is inextricable from human health. The climate crisis highlights the need for emergency preparedness for climate-related disasters, sustainable health systems that can withstand these challenges, and a broader understanding of the links between climate and health. Health professionals must recognize that the health of the environment is intrinsic to human health and must seek to influence as individuals, a collective, and on a policy level in the interests of all humanity [4].

The International Council of Nurses, the World Health Organization, the Sigma Theta Tau, and the Alliance of Nurses for Healthy Environments recognize that nurses have great potential to enact climate protective actions [1]. Nurses have three essential assets in this area [1]. First, they constitute about 60% of health professionals worldwide. Second, nurses are trusted, as they are often the first health care provider people encounter when seeking care. Third, they are close to the people most vulnerable to climate change. Nurses are critical to achieving the sustainable development goals that, like the planetary health framework, focus on environmental sustainability and human well-being. Rosa et al. [5] also proposed a shift from “global nursing,” which describes nursing’s role in global health endeavors, toward “planetary nursing,” which invites broader possibilities for nurses to engage in worldwide health initiatives related to planetary health. Nurses, as leaders, should consider their own health system environments for each of the direct, indirect and ecosystem-mediated health factors, and then outline impacts and develop anticipatory plans [6].

Although national nursing organizations have addressed the importance of education and advocacy, many nursing students are not adequately prepared regarding the health impacts of climate change and nursing profession response [7]. To reduce emissions and meet the 2030 Sustainable Development Goals (specially Goal 13, Target 3: “Improving environmental education and awareness”), nursing education must equip undergraduates with the knowledge and skills they need to sustainably promote the health and well-being of current and future generations, as well as that of the planet [8]. It is necessary to equip students with knowledge about the health risks posed by climate change and how to prevent these problems through primary prevention strategies, as well as providing them with the skills to make responsible use of resources both in their work and on a personal level, as health workers serve as role models for the population. Environmental sustainability and stewardship need to be taught so that nurses and students not only understand but also have the ability to initiate change [1]. Therefore, embedding planetary health education in curricula is an essential step to achieving the transformative change needed [2, 9]. A full explanation of the competencies to be included in the nursing programs is shown in Kelly and Lazenby [10]. While, Schwerdtle et al. [11] developed 12 tips for teaching environmental sustainability to health professionals.

Mainstreaming climate change teaching and learning is important in all stages of education, but Higher Education Institutions have an essential role. Thew et al. [12] state that the mainstreaming of this topic in curricula must achieve an education that ensures that all students and staff members are engaged with climate change. Clinical educators at faculty level have generally resisted the inclusion of planetary health in their undergraduate and postgraduate curricula [13]. Shea et al. [14] found that 74% of the higher education institutions surveyed reported that the addition of climate-health offerings is still under discussion. The integration of climate change into nursing education is essential and requires that knowledge, skills, and insights critical for clinical practice in our climate-changing world are incorporated in curricula, practice, research, and policy [7]. Global warming and climate change are sensitive and important issues that cannot be handled and evaluated only at the level of knowledge, as only the knowledge of these topics is not related to the awareness, attitudes, and behaviors of nursing students [3].

Interactive scenarios have enabled students to improve their awareness, attitudes, and behaviors on the health effects of climate change beyond knowledge acquisition. The University of Plymouth, has embedded sustainable healthcare education into the nursing curriculum to strengthen clinical practice and leadership skills, using the free, online evidence-based sustainability literacy and competency resources in nursing education (NurSusTOOLKIT) (http://nursus.eu/). The use of case studies, focus groups, role plays, simulations, and skill sessions with sustainability and health scenarios is used to work with multidisciplinary groups discussing sustainability issues [15]. In addition, pedagogies such as problem-based, practice-based, enquiry-based, and project-based learning which encompass the real-life challenges of climate change and places students at the center of identifying and evaluating solutions, can help facilitate interdisciplinary learning [12]. Using augmented reality (AR) also helps to enrich educational scenarios with more visual, audio, and virtual information. AR is being incorporated into the education sector because of its ability to visually convey abstract concepts and provide 3D information with real objects. AR enables active and participatory learning by enriching real educational scenarios, increasing student motivation, and, as a complement to clinical simulation, is effective for acquiring nursing competencies and is seen as an opportunity to promote student-centered learning [16]. Simulation practice using augmented reality has been shown to be effective for psychomotor skill acquisition in nursing students [11, 13]. However, there is no evidence of the use of augmented reality for environmental health in higher education in nursing.

Therefore, this study aimed to: i) assess the contribution of scenario-based learning and augmented reality to environmental awareness and attitudes toward climate change and sustainability among undergraduate nursing students, ii) determine the extent to which undergraduate nursing students apply their competencies in sustainability in clinical practice, and iii) assess students’ perception of the appropriateness of the scenario-based learning approach to their learning and its usefulness in engaging students to make a positive change within their clinical practice.

## Methods

### Study design and participants

A time-series analysis study was designed to assess nursing degree students throughout their four-year academic university program using scenario-based learning and augmented reality related to sustainability, climate change, and health. The target population comprised all enrolled undergraduate first-year nursing students in a Spanish school of Nursing, University of Jaén (academic year 2019-2020). The inclusion criteria only stated that these students consented to participate. Later, in the academic years 2020-2021 and 2021-2022, this purposive sample participated in teaching sessions specially designed to create environmental awareness and develop competencies in sustainability, climate change, and health. The sample size was calculated based on the results of previous researches [17, 18], to detect a difference of 1 point at the *Sustainability Attitudes in Nursing Survey*, standard deviation of 5.5 points at each group, confidence level of 95% and a minimum power of 80%, being the minimum sample size 81 pairs.

All students received information regarding the study and signed consent. Participation was voluntary, and students had the right to withdraw without adverse effects on their academic standing. The confidentiality and anonymity of personal data were maintained by coding students’ identity. The surveys required this code to match them with the completed surveys in the following academic years. Teachers were not aware of specific student responses. The data collected were used for specific research purposes and kept in the custody of the researchers.

### Procedures

Students starting their Nursing degree in September 2019 were invited to participate in this study. Later, the same students participated in three respective sessions (one in the second year of the degree and two in the third year). All the learning materials used are available at: http://nursus.eu/ (Erasmus + KA2 Project, 2014-2017). Hence, the nursing students were asked to complete the questionnaires in four occasions:

- At the start of academic Year 1 (in 2019), before students had any exposure to sustainability teaching (to avoid reporting bias).
- In Year 2: after a scenario-based session about an asthmatic child exposed to pollutants at home, school, and city, whose health and care were compromised by the housing conditions and environment in which he lives. The case study was based on real circumstances of the effects of climate change and problems arising from excessive industrialization. (Based on Topic J3_A1 NurSus). Session 1.
- In Year 3:
  - ○ After a scenario-based session about waste management related to bladder catheterization, which reflected a healthcare treatment with high environmental impact; a nurse performing this procedure does not properly use infection prevention materials, discards unused open material, and does not recycle the large amount of waste generated. Students should analyze the relationship between the prevention of urinary tract infection and the rational use of material, proper waste separation, environmental, and economic costs of healthcare waste management. (Based on Topic P2_B1, NurSus). Session 2.
  - ○ After a session about an older person who is poly-medicated, multi-pathological, mobility impaired, and dependent in need of a carer. Due to high summer temperatures and a 3-day heatwave, the older person shows signs and symptoms of dehydration. In this simulation, the students should analyze the consequences of climate change on the health of this population group, which is vulnerable to temperature changes. (Based on Topic E3_B2, NurSus). Session 3.

Additionally, during these sessions, augmented reality facilitated the deep “immersion” of the students in the clinical situation with more realistic visualization. The linked digital content (3D images and videos) were obtained from the applications MOZAIK education®, Sketchfab®, Biodigital®, and MERGE®. As markers to activate digital information, QR codes and MERGE cubes were used to increase the learning potential in a specific scenario. Students could access the augmented reality integrated into the learning scenarios created for each session using tablets or mobile phones.

Data were collected using self-administered questionnaires through an online tool (Survey Monkey) in computer rooms or using their own laptops or mobile phones during mandatory training classes (there were a maximum of 15 students per session and the duration of each one was 150 minutes). The questionnaires included the following:

The *Sustainability Attitudes in Nursing Survey* (SANS_2) was used to measure nursing students’ attitudes toward climate change and sustainability. SANS_2 comprises five items whose response options range from 1 (strongly disagree) to 7 (strongly agree) on a Likert-type scale, with a maximum score of 35 points. Reliability analysis showed a Cronbach’s alpha of 0∙82, and the five items loaded on a single factor explained 58% of total variance [17]. It was subsequently amended to capture attitudes to sustainability practice ‘at home’ as new students (year 1) were unlikely to be able to report sustainability in clinical practice. Content validity was again determined through discussions with nursing education and sustainability experts, and they rated content to assess the desired construct [19]. Level of attitudes were categorized in: Excellent (scores > 90%), Very good (scores 70–89%), Good (scores 50–69%), Not enough (scores 30–49%) and Poor (scores < 29%) [20].

In addition, several items (6–8) were added to investigate sustainability awareness of nursing students in the first year (Table 1) and items 9–12 to investigate sustainability awareness in nursing practice in the third year after completing their first clinical placement (336 hours during 6 weeks) in healthcare institutions (Table 2).

**Table 1:** Sustainability attitudes (items 1-5) and awareness (items 6-8) in nursing survey

1. Climate change is an important issue for nursing. 2. Issues about climate change should be included in the nursing curriculum. 3. Sustainability is an important issue for nursing. 4. Sustainability should be included in the nursing curriculum. 5. I apply sustainability principles at home. 6. All university students should learn about the impact of climate change when studying their subject. 7. Concerns about the environment influenced my choice of university. 8. The University of _________‘s sustainability reputation influenced my choice of university.

**Table 2:** Awareness in nursing practice items

9. I apply sustainability principles in my nursing practice. 10. I am aware of unsustainable practice in my work environment. 11. I challenge unsustainable practice in my work environment. 12. I feel unable to challenge unsustainable practice in my work environment.	

Students were also asked questions about the usefulness of the teaching sessions and the scenarios presented to examine the perceived reality and relevance of the pedagogical approach and content (Table 3). Responses were on a seven-point scale where 1 was strongly disagree and 7 was strongly agree. Content validity was determined through discussions with nursing practice education and sustainability experts, and they rated content to assess the desired construct. The questionnaires shown below were already used in a previous study at Plymouth University [21].

**Table 3:** Questions for feedback on the sustainability scenario-based sessions

1. The scenario was realistic. 2. The resources were useful. 3. The session was interesting. 4. The session was engaging. 5. The session helped me to make links between climate change and health. 6. I enjoyed the session. 7. The session helped me to make links between resources and heath. 8. I would prefer this session as a lecture.	

**Fig. 1:**
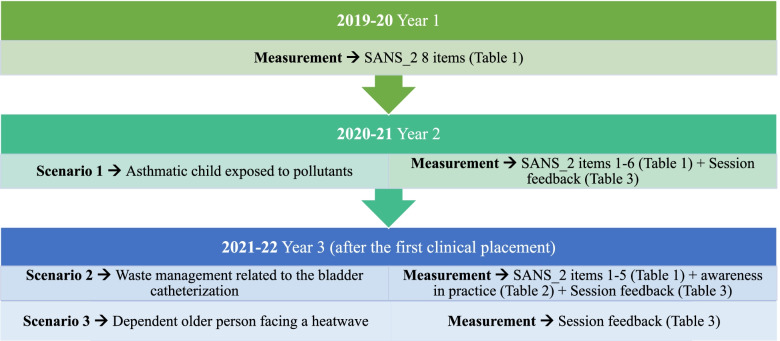
Summary of the sessions and data collection in each academic year

Figure 1 shows a summary of the procedures.

### Statistical analysis

Questionnaire data are presented as mean and standard deviation: SANS_2 mean value (mean items 1 to 5) and individual items (1–12) per year. Reliability was calculated by Cronbach alpha. Kolmogorov-Smirnov test for distribution for all variables was significant (*p* < 0∙001), indicating non-normal distribution. SANS_2 mean and total score and items 1–5 were compared for surveys completed in the 3 years; all feedback items were compared for surveys completed in year 2 and twice in year 3. Only data from cases that completed all measurements required for each analysis were analyzed. Related sample Friedman and Wilcoxon signed-rank tests were used to test for differences in survey scores for different times (years). The level of significance was established at 0∙05. All analyses were performed using SPSS version 25.

### Ethical approval and consent

This study was granted by the Research Ethic Committee of University of Jaen (JUL.19/3.PRY) and was performed in accordance with the ethical standards laid down in the Declaration of Helsinki. All students were informed and gave consent to participation in the study. The anonymity and confidentiality were guaranteed. No risks were foreseen in participating in this study, but any participant could leave the study in any time.

## Results

The sample in the first year included 119 nursing students (of 140 enrolled); however, only 96 completed the questionnaires all four times (19.3% lost). Demographic data are reported in Table 4.

**Table 4:** Demographic data of the sample (*N* = 96)

Demographic data	n (%)
Age (M/SD)	21.13 (4.82)
Gender	
Male	23 (24)
Female	73 (76)
Pathway to University	
Baccalaureate	52 (55.2)
Professional training	37 (38.5)
Other university degree	2 (2.1)
Over 25 years old	3 (3.2)
Over 45 years old	1 (1)
Attended a sustainability session	
Yes, within the prior three months	29 (30.2)
No	54 (56.3)
Yes, more than 3 months prior	13 (13.5)

The SANS_2 scale (items 1–5) showed good reliability (0∙78 first year, 0∙87 second year, and 0∙74 third year). Likewise, the reliability of the whole questionnaire (items 1–8) was good (0.80) in the first year.

### Environmental attitudes and awareness toward climate change and sustainability

Questionnaires were matched to ensure that the same participants were compared across the 3 years of their nursing education. A total of 101 questionnaire matched pairs were compared for years 1 and, 113 pairs were compared for years 2 and 3, and 98 pairs were compared for years 1 and 3. Significant differences were found between the first and second and between the first and third years for the SANS_2 mean and total score and for each item except item 5 (*I apply sustainability principles at home*). No significant differences were found for any item or mean between the second and third years (Table 5). Item 6 (*All university students should learn about the impact of climate change when studying their subject*) increased from the first to the second year (6.336) but without significant differences. The scores for items 7 and 8 in the first year were low (1.5 and 1.54 respectively).

**Table 5:** Comparisons of statements among Years 1, 2, and 3

Statement	Year 1 (***N*** = 120)	Year 2 (***N*** = 131)	Year 3 (***N*** = 122)
1. Climate change is an important issue for nursing.	6.05	6.702^a^	6.754^b^
2. Issues about climate change should be included in the nursing curriculum.	5.1	6.176^a^	6.246^b^
3. Sustainability is an important issue for nursing.	5.792	6.458^a^	6.574^b^
4. Sustainability should be included in the nursing curriculum.	5.033	6.053^a^	6.189^b^
5. I apply sustainability principles at home.	5.3	5.344	5.361
Mean	5.4729	6.1583^a^	6.2250^b^
Total	27.365	30.792^a^	31.125^b^

^a^Significant differences between Year 1 and 2

^b^Significant differences between Year 2 and 3

The excellent level of attitudes increased in years 2 and 3 with respect to year 1; however, the other levels decreased (except poor level; Table 6).

**Table 6:** Number (and percentages) of participants according to the level of attitudes

Academic Year	Excellent	Very good	Good	Not enough	Poor
Year 1	20 (20.8)	56 (58.3)	15 (15.6)	5 (5.3)	0
Year 2	55 (57.3)	36 (37.5)	2 (2.1)	1 (1)	2 (2.1)
Year 3	54 (56.3)	38 (39.6)	3 (3.1)	0	1 (1)

### Awareness in clinical practice

Awareness in clinical practice was assessed in third-year students after completing their first 6-week clinical placement. Nursing students scored the lowest for item 12: *I feel unable to challenge unsustainable practice in work environment* (3.639), item 9: *I apply sustainability in my nursing practice* (4.426), and item 11: *I challenge unsustainable practice in work environment* (4.545). However, scored the highest for item 10 about awareness: *I am aware of unsustainable practice in my work environment* (6.233).

### Feedback on the relevance and usefulness of the scenario-based approach

Reliability values for the feedback survey were good (Cronbach’s alpha ranges from 0.84-Session 3 to 0.92-Session 1). All items were scored above 6 except item 8 (*I would prefer this session as a lecture*). Significant differences were found for items 6 (*I enjoyed the session*) and 8 (*I would prefer this session as a lecture*) in year 3 between Session 2 and Session 3 (Table 7).

**Table 7:** Session feedback mean scores

Statement	Year 2 (***N*** = 131)	Year 3 (***N*** = 122)	
	Session 1	Session 2	Session 3
1. The scenario was realistic.	6.344	6.25	6.3
2. The resources were useful.	6.420	6.42	6.43
3. The session was interesting.	6.366	6.43	6.38
4. The session was engaging.	6.176	6.39	6.21
5. The session helped me to make links between climate change and health.	6.305	6.48	6.37
6. I enjoyed the session.	6.191	6.46	6.19^a^
7. The session helped me to make links between resources and heath.	6.282	6.48	6.31
8. I would prefer this session as a lecture.	4.435	4.04	4.83^a^

^a^Significant differences between Session 2 and Session 3

Feedback on the relevance and usefulness of the scenario-based approach showed that more than 90% of participants rated positively the Sessions 1 and 3 and more than 93% the Session 2. For the statement *I would prefer this session as a lecture* only 40% in Session 2 agreed (scored more than 5 on the seven-point scale). In line with the finding that around 95% of participants or more (in all years) felt that the design of the sessions had helped them make links between climate change and health (point 5; Table 8).

**Table 8:** Percentage of scores above 5 on session feedback

Statement	Year 2 (***N*** = 131)	Year 3 (***N*** = 122)	
	Session 1	Session 2	Session 3
1. The scenario was realistic.	94.7	93.4	93.9
2. The resources were useful.	96.2	94.2	95.7
3. The session was interesting.	93.1	94.3	93
4. The session was engaging.	90.8	93.4	90.4
5. The session helped me make links between climate change and health.	95.4	97.5	94.8
6. I enjoyed the session.	90.8	96.7	92.1
7. The session helped me make links between resources and heath.	93.1	98.4	91.3
8. I would prefer this session as a lecture.	51.9	40.2	55.7

## Discussion

This study investigated environmental awareness and attitudes toward climate change of nursing students and the contribution of scenario-based learning and augmented reality to these. Following a training program using scenario-based learning and augmented reality related to sustainability, climate change, and health in the Nursing degree, more than 95% of the students in years 2 and 3 have excellent or very good attitudes. The present study observed a significant increase in overall attitudes, and for four of the five items, from year 1 to 2 and from year 1 to 3. As their education increases, attitudes improved toward the importance of climate change and sustainability in their education as nurses, a finding in line with those of other authors [22].

In order to increase environmental awareness and attitudes, topics such as waste management or sustainable use of resources during the performance of nursing practice should be incorporated in the training programs of the nursing degree. Sessions on sustainability, climate change, and health for nursing students has been implemented to highlight their relevance in professional practice and increase environmental awareness and attitudes to take action against climate change and its effects on health [3, 18, 22]. Making changes in nursing curriculums will not only increase nursing’s leadership capacity, but also make nursing boards and licensing councils reconsider the importance of fundamental climate change knowledge as a prerequisite for licensure [1]. It is a priority to mainstream climate change education to prepare students for their roles in work and the wider society, now and in the future. The complexity of the climate crisis means all disciplines have a role to play [12], and our study and another study [21] show that all university students should learn about it. However, despite the increased visibility of sustainable and healthy universities promoting its inclusion in their curricula [23], this does not seem to influence students’ choice of university.

We found that students in upper grades had higher self-perception of their attitude to influence unsustainable practices than students in lower grades because of their greater environmental awareness. That students in lower grades showed less positive attitudes, along with reasons such as lack of confidence, afraid to challenge, lack of knowledge, or resistance to change, among others, may explain the inability to question unsustainable practices or the failure to change attitudes [22]. In comparison, after completing a clinical placement, the third year students presented a high awareness of unsustainable practices in their work environment. However, they were less able to apply sustainability principles in their nursing practice and, above all, feel unable to challenge unsustainable practices in their work environment. As trainees, they do not have sufficient authority to establish changes in practice as they are under the tutelage of their mentors. Hence, they have greater difficulty applying sustainability principles to nursing practice despite having a greater awareness of the environmental impact on their nursing practice. The main barriers to challenging unsustainable practice are lack of confidence and resistance to change. Literature reports this lack of confidence to stem from lack of knowledge, being afraid to challenge, and power of imbalance/hierarchy because they are still students and not professionals [22]. However, this did not happen with the item on applying sustainability principles in the home, for which there is no such increase in scores and no differences by years. It is likely that the training received in this program does not influence attitudes at home. The program subject matter was oriented to the relationship between climate change, sustainability, and health, whereas the home is a different environment of influence from the professional environment.

The scenario-based approach to teaching is ideally suited to increase the development of environmentally sustainable nursing practice. Students rate the usefulness of the scenarios highly, noting that the session helped them make links between climate change and health, and between resources and health. Learners are more likely to face unsustainable practices in the work environment after participating in scenario-based learning [24]. The use of a scenario-based learning approach with nursing and midwifery students can modify attitudes and knowledge toward sustainability and climate change. Incorporating this approach in the context of clinical skills provides a current and engaging approach that is both educationally sound and clinically relevant [25].

More than 90% said they enjoyed the sessions and scored low when asked if they would prefer this session as a lecture. The additional use of Augmented Reality resources may have increased the value given owing to the realism of the scenarios, and the novelty of the new teaching technologies may have influenced students to prefer the scenario-based approach. Augmented reality helps to bring the student closer to the reality that surrounds them in a more concrete and experiential way to understand the world in which he lives [26]. A combination of teaching approaches and methods to develop sustainability awareness in health professions students is better than a single methodology [24]. Hence, the combination of augmented reality and scenario-based approach to learning used in this research made students more receptive to the training. Virtual reality and augmented reality technologies offer a great opportunity to revolutionize nursing education and promote student-centered learning [16]. Virtual reality simulation is the most effective in improving students’ cognitive [27] and affective outcomes on evidence-based practice [28].

The main methodological limitation of the study is that changes in awareness and attitudes scores cannot be linked to the use of augmented reality and scenarios, due to the fact that there was no control group to compare with, since it does not appear ethical to leave one group of students without high-quality education in this important subject. Other limitation of this study is the rate of student losses during the follow-up; during the 3 years of the nursing degree, there are student transfers and mobility grants to other universities and also new students who could not be surveyed at the beginning of the cohort. Another limitation to consider is the use of training with three different scenarios in each course, with different learning competencies. Although the same methodological structure was followed to facilitate temporal comparisons. Finally, the study was conducted in only one university.

As future lines of research, we propose to continue evaluating students’ attitudes and environmental awareness after completing all clinical placements, especially to assess competencies in challenging the environmental impact of practices they encounter during their training. In addition, it is necessary to design and conduct qualitative studies to explore students’ experiences and perceptions when applying these environmental competencies in the clinical setting.

In conclusion, the scenario-based learning and augmented reality increase environmental awareness and attitudes toward climate change and sustainability among nursing students. Students have a high awareness of unsustainable practices in their work environment but have difficulty applying sustainability principles to nursing practice. Students rated the scenario sessions very positively, noting that it helped them make links between climate change and health, and between resources and health.

## Supplementary Information

**Additional file 1.** Database

## Data Availability

The datasets supporting the conclusions of this article are included within the article (and in the Additional file 1).
